# Синдром поликистозных яичников: новые и перспективные варианты лечения

**DOI:** 10.14341/probl13400

**Published:** 2024-09-16

**Authors:** Д. М. Гасиева, Е. В. Шереметьева, М. Ф. Калашникова, Ф. Х. Дзгоева, Е. Т. Алборова

**Affiliations:** Национальный медицинский исследовательский центр эндокринологии; Национальный медицинский исследовательский центр эндокринологии; Институт клинической медицины им. Н.В. Склифосовского Первого МГМУ им. И.М. Сеченова (Сеченовский Университет); Национальный медицинский исследовательский центр эндокринологии; Национальный медицинский исследовательский центр эндокринологии

**Keywords:** СПЯ, диетотерапия, инозитол, витамин D, витамин Е

## Abstract

Синдром поликистозных яичников (СПЯ) — полигенное эндокринное расстройство, обусловленное как генетическими, гормональными, так и эпигенетическими факторами. Во всем мире актуальность проблемы СПЯ обусловлена распространенностью синдрома от 10 до 13%, а также развитием сопряженных с ним состояний, негативно влияющих на здоровье и жизнь женщины: бесплодие, дерматологические проявления гиперандрогении (гирсутизм, угревая болезнь), сердечно-сосудистые патологии, метаболические и психоэмоциональные нарушения. В настоящее время предложено много теорий развития данного заболевания, и как следствие — методов воздействия на него и лечения. Согласно клиническим рекомендациям Минздрава РФ, в основу комплексной терапии положена модификация образа жизни пациентки. Мы провели анализ около 60 статей, посвященных различным диетологическим подходам к лечению СПЯ. Поиск статей проводился на базе PubMed, Nature reviews, Oxford academic, Clinical nutrition, EJOG, Science Direct, MDPI. Лучше всего себя зарекомендовала средиземноморская диета, DASH-диета, кетогенная диета и низкоуглеводная. Также не стоит игнорировать необходимость добавления в рацион женщины витаминов D и Е, фолиевой кислоты, кальция, различных про- и пребиотиков. Перспективным методом терапии СПЯ в настоящее время становится применение инозитола и препаратов ГПП-1. Согласно результатам проведенного анализа, отмечено значительное влияние диетотерапии на антропометрические и биохимические показатели женщины. Основное, что мы хотим сказать: в подходе к лечению пациенток с СПЯ стоит учитывать индивидуальные особенности каждой и не ограничиваться только медикаментозной терапией.

## Abstract (en)

Polycystic Ovary Syndrome (PCOS) — is a polygenic endocrine disorder caused by genetic, hormonal, and epigenetic factors. The importance of addressing PCOS lies in its prevalence, affecting approximately 10 to 13% of women, as well as the associated health conditions that can have a negative impact on women’s lives, including infertility, dermatological manifestations of hyperandrogenism (such as hirsutism and acne), cardiovascular pathologies, metabolic disorders, and psychoemotional disorders. Currently, many theories of the development of this disease have been proposed, and as a consequence of methods of influencing and treating it. According to the clinical recommendations of the Ministry of Health of the Russian Federation, modifying the patient’s lifestyle forms the basis of comprehensive therapy. We have analyzed about 60 articles on various dietary approaches to the treatment of PCOS, sourced from PubMed, Nature Reviews, Oxford Academic, Clinical Nutrition, EJOG, ScienceDirect, and MDPI. The Mediterranean diet, the DASH diet, the ketogenic diet and the low-carb diet have proven themselves the best. Also, it’s necessary to add vitamin D, E, folic acid, calcium, various pro- and prebiotics to a woman’s diet. Currently, the use of inositol and GPP-1 is becoming a promising method of PCOS therapy. According to the results of the analysis, a positive effect of diet therapy on the anthropometric and biochemical parameters was noted. Based on the analysis, a positive effect of diet therapy on anthropometric and biochemical parameters has been observed. It is crucial to consider the individual characteristics of each patient and not rely solely on drug therapy when approaching the treatment of PCOS.

Мы благодарим рецензентов за тщательный анализ нашей статьи и за высказанные критические замечания. Замечание по поводу УЗ-признака СПЯ принято во внимание и исправлено.

Синдром поликистозных яичников (СПЯ) — полигенное эндокринное расстройство, обусловленное генетическими, гормональными и эпигенетическими факторами. В соответствии с клиническими рекомендациями Минздрава РФ и критериями объединенной рабочей группы экспертов ЕSHRE/ASRM (2003 г.), для постановки диагноза СПЯ должны быть выявлены два Роттердамских критерия из трех при отсутствии какой-либо иной причины синдрома. Это гиперандрогения (биохимическая и/или симптоматическая); олиго- и ановуляция; поликистозная структура яичников: УЗИ-признак, рекомендовано опираться на ультрасонографические критерии с применением трансвагинальных датчиков: наличие ≥20 фолликулов диаметром 2–9 мм в любом яичнике и/или увеличение объема любого яичника ≥10 см³, при использовании датчиков с меньшим разрешением или при трансабдоминальном исследовании увеличение любого яичника ≥10 см³, при отсутствии желтого тела, кист или доминантных фолликулов [1][2].

Согласно данным обновленного международного клинического протокола по СПЯ (2023 г.), это наиболее распространенная эндокринопатия (от 10 до 13%), поражающая женщин репродуктивного возраста, а также трансформирующаяся на протяжении всей жизни: от менархе до постменопаузы — и приобретающая те или иные гинекологические (бесплодие), метаболические, сердечно-сосудистые, психологические аспекты [1][3][4][5].

## ПАТОГЕНЕЗ

Несмотря на многочисленные исследования со времен Штейна и Левенталя до настоящего времени, так и не удалось сформулировать единую фундаментальную концепцию патогенеза СПЯ. На сегодняшний день предложено несколько теорий развития этого заболевания.

К основным патогенетическим механизмам относят гиперандрогению, инсулинорезистентность (ИР), избыточную массу тела или ожирение. Затруднительно утверждать, что именно становится «пусковым» механизмом: сложность в определении единой первопричины возникновения СПЯ связана с индивидуальными генетическими и фенотипическими характеристиками каждой пациентки [6].

Если рассматривать избыточное накопление жировой массы как одну из основных причин развития СПЯ, необходимо учитывать, что ведущую роль играет именно висцеральная жировая ткань [7]. По мере накопления жировой ткани, особенно висцеральной, увеличивается содержание в крови циркулирующих свободных жирных кислот. Они используются скелетной мускулатурой в качестве источника питания вместо глюкозы, что приводит к гипергликемии, которая в свою очередь стимулирует компенсаторную гиперинсулинемию, оказывающую патологическое стимулирующее влияние на рецепторы к лютеинизирующему гормону (ЛГ) в текальных клетках и в стромальной ткани яичника.

Еще один патогенетический механизм СПЯ связывают с одним из адипокинов — лептином, пептидным гормоном, регулирующим энергетический обмен, и также способным ингибировать экспрессию ароматазной мРНК в клетках гранулезы, что угнетает ароматизацию андрогенов в эстрогены и в дальнейшем ведет к развитию овариальной гиперандрогении [8].

Инсулинорезистентность может быть как следствием висцерального ожирения, так и самостоятельной, первично возникшей причиной патогенеза СПЯ. Инсулин влияет на выработку андрогенов и формирование поликистозной структуры яичников, на активность фермента Р450с17 и взаимодействует с ЛГ [9], что в совокупности может приводить к гиперандрогении. Инсулин может воздействовать и на уровень глобулина, связывающего половые гормоны (ГСПГ), снижая его концентрацию, что опять же ведет к увеличению продукции тестостерона в яичниках. Инсулин также стимулирует липогенез, ингибирует липолиз, что способствует дальнейшему накоплению жировой ткани [7][10].

Также возможно существование идиопатической гиперандрогении на фоне ферментативной активности. Существует теория, по которой тестостерон может превращаться в дигидротестостерон не только под действием фермента 5-альфа-редуктазы, но и 5-альфа-редуктазы 1/2, α-кеторедуктазы типа 1C2 и 17β-HSD6. Андрогены могут влиять на накопление висцеральной жировой ткани, тем самым запуская все вышеперечисленные механизмы СПЯ. Таким образом, мы наблюдаем формирование порочного круга патологических процессов у женщин с изучаемым синдромом [7].

Помимо вышеперечисленных классических патогенетических механизмов развития СПЯ, в настоящее время одним из актуальных направлений в исследованиях может стать изучение ферментов, влияющих непосредственно на процесс овуляции. Пик ЛГ активирует ферменты из семейства металлопротеиназ (MMP2, 9, 14, 19), ADAMTS-1, необходимых для овуляции. При обследовании женщин, перенесших экстракорпоральное оплодотворение (ЭКО), было выявлено снижение ММРS2 и ММРS9 в фолликулярной жидкости и увеличение содержания ингибитора металлопротеиназ ТIMРs-1. В настоящее время ведущая роль отдается протеазам из семейства ADAMTS, так как перед овуляцией экспрессия ADAMTS1 резко возрастает, приводя к деградации внеклеточного матрикса с последующим разрывом фолликула, тогда как аномальная экспрессия гена может наоборот приводить к нарушению разрыва фолликула и тем самым способствовать ановуляции с последующим формированием кистозной атрезии фолликулов [11][12].

Таким образом, учитывая значительную гетерогенность заболевания, в настоящее время, согласно клиническим рекомендациям Минздрава РФ и международному научно-обоснованному руководству по оценке и лечению СПЯ [13], в основе комплексной терапии СПЯ должна быть модификация образа жизни, учитывающая диетологические и нутритивные аспекты воздействия на здоровье женщины.

## ДИЕТОЛОГИЧЕСКИЙ ПОДХОД В КОМПЛЕКСНОЙ ТЕРАПИИ СПЯ

В связи с важной ролью диетологического аспекта в лечении СПЯ рассмотрим исследования, в ходе которых изучалось влияние различных диет на маркеры СПЯ.

Метаанализ восьми рандомизированных контролируемых исследований (РКИ) с участием 327 пациенток показал, что низкоуглеводная диета, особенно долгосрочная (более 4 недель), положительно влияет на течение СПЯ. В результате Z. Xiaoshuai et al. отметили уменьшение ИМТ, HOMA-IR, тестостерона, липидов крови и увеличение ФСГ, ГСПГ [14].

В исследовании A. Paoli et al. оценивалось влияние кетогенной диеты, которой придерживались 14 женщин с СПЯ в течение 12 недель. Улучшение было отмечено по всем антропометрическим, биохимическим и гормональным показателям [15].

Помимо низкоуглеводной, активно изучается влияние средиземноморской диеты на здоровье женщин в различных когортах как среди европеоидного, так и азиатского населения. L. Barrea et al. продемонстрировали, что средиземноморская диета, богатая сложными углеводами, клетчаткой, полиненасыщенными жирами, биофлавоноидами и макро- и микроэлементами, оказалась наиболее подходящей для женщин с СПЯ. В исследовании приняли участие 224 женщины европеоидной расы: 112 пациенток с СПЯ и 112 здоровых женщин составили контрольную группу. Было выявлено, что пациентки с СПЯ вообще употребляют в пищу меньше оливкового масла, бобовых, рыбы/морепродуктов и орехов, чем участницы контрольной группы. Данное наблюдение позволило сделать вывод, что развитие СПЯ коррелирует с составом продуктов питания [16].

Как ни парадоксально, но вполне убедительно у женщин с СПЯ зарекомендовала себя DASH-диета (The Dietary Approaches to Stop Hypertension). Отличие ее от других низкокалорийных диет в том, что она богата фруктами, овощами, нежирными молочными продуктами и резко ограничивает потребление продуктов с высоким гликемическим индексом. Придерживаясь DASH-диеты, человек потребляет большое количество пищевых волокон и антиоксидантов: витаминов (фолиевая кислота и др.) и минералов (магний, кальций, железо, селен и др.), что может положительно влиять на аномальный метаболический профиль и ИР у женщин с СПЯ.

В РКИ, проведенном в течение 12 недель, участвовали 60 пациенток: 30 в группе DASH-диеты и 30 — в контрольной. В группе DASH отмечалось снижение ИМТ, антимюллерова гормона (АМГ) и увеличение ГСПГ [17][18].

Ниже приводим сводную таблицу данных, полученных в нескольких исследованиях (табл. 1).

На основании результатов исследований, приведенных в табл. 2, можно заключить, что диетотерапия оказывает существенное положительное влияние на течение СПЯ, включая улучшение биохимических и антропометрических показателей. Однако, несмотря на необходимое количество потребленных макронутриентов, ученые обнаружили в питании женщин с СПЯ дефицит микронутриентов, связанный с недостатком пищевых волокон, витаминов D и Е. Пациентки также имели более низкие уровни ГАМК-продуцирующих бактерий, которые могут ухудшать течение СПЯ через ось «микробиота кишечника–мозг» [19][20].

**Table:** Таблица 1. Влияние различных диет на течение СПЯ

Вид и продолжительность диеты	Численность исследованных	Авторы и год публикации	Результаты
Низкоуглеводная диета: в течение более 4 недель	8 РКИ;n=327 женщин с СПЯ	Xiaoshuai Zhang et al., 2019 г. [14]	ИМТ, HOMA-IR, тестостерон ↓ФСГ, ГСПГ ↑
Кетогенная диета: в течение 12 недель	N=14 женщин с СПЯ	Paoli A. et al., 2020 г. [15]	ИМТ, тестостерон,масса тела ↓ФСГ, ГСПГ↑
Средиземноморская диета. Приверженность ей оценивалась по данным дневника питания за 7 дней и анкетирования из 14 пунктов	N=224 женщин;n=112 с СПЯ; контрольнаяn=112	Barrea L. et al., 2019 г. [16]	Пациентки с СПЯ употребляют меньше продуктов, содержащих ПНЖК
DASH-диета: в течение 12 недель	РКИ; n=60 женщин с СПЯ	Foroozanfard F. et al., 2017 г. [17]	ИМТ, АМГ↓ГСПГ ↑

Существует немало исследований, показывающих взаимосвязь дисбактериоза кишечной флоры и СПЯ. В исследовании 163 женщин с СПЯ или поликистозом яичников, по данным УЗИ, была обнаружена корреляция между СПЯ и микробиотой: у пациенток с СПЯ микробиота была менее разнообразна [21].

L. Lindheim et al. в своем исследовании также продемонстрировали, что при СПЯ наблюдается снижение как разнообразия микробиоты, так и барьерной функции кишечника, а также наличие эндотоксемии [22]. Основываясь на результатах данных наблюдений, следует рассмотреть целесообразность введения в рацион женщин с СПЯ пробиотиков.

## РОЛЬ ПРОБИОТИКОВ И ПРЕБИОТИКОВ В ДИЕТОТЕРАПИИ СПЯ

Пробиотики — живые микробные пищевые добавки, которые формируют и уравновешивают содержание различных кишечных бактерий хозяина. Функции пробиотиков разнообразны; в данном контексте актуальны защита кишечного барьера, улучшение чувствительности к инсулину и регулирование иммунной системы [23].

Метаанализ 17 рандомизированных контролируемых исследований с 1049 участниками показал, что прием пробиотиков может положительно влиять на метаболические показатели, такие как липопротеины высокой плотности (ЛПВП), триглицериды и инсулин натощак, но не оказывает очевидного влияния на массу тела, HOMA-IR, окружность талии [24]. Кроме того, сообщалось, что комбинация пробиотиков с другими веществами, такими как антиоксидантны, селен и витамин D, в лечении СПЯ также имеет положительное влияние на течение заболевания (табл. 2) [25][26].

**Table:** Таблица 2. Влияние пробиотиков и селена на уровень тестостерона и ГСПГ [25]

Общий тестостерон (нг/мл)	1,3±0,5	1,3±0,4	1,4±0,7	1,1±0,6	−0,26(−0,51; −0,02)	0,03
ГСПГ (нмоль/л)	40,3±17,5	40,4±18,3	47,1±19,7	49,5±22,1	1,82(−1,77; 5,42)	0,31

Влияние пробиотиков на течение СПЯ изучали еще в 2015 г. T. Shoaei et al. В течение восьми недель 32 пациентки принимали пробиотики Lactobacillus casei, Lactobacillus acidophilus, Lactobacillus rhamnosus, Lactobacillus bulgaricus, Bifidobacterium breve, Bifidobacterium longum и Streptococcus thermophiles. В результате проведенного лечения было отмечено снижение уровня глюкозы плазмы, а также положительное влияние на снижение среднего уровня инсулина и HOMA-IR (табл. 3) [27].

**Table:** Таблица 3. Влияние пробиотиков на уровень глюкозы натощак, инсулина, HOMA-IR [27]

Лабораторные показатели	Плацебо(n=33)	P	Пробиотики(n=32)	Р
Глюкоза натощак (мг/дл)Неделя 0Неделя 8Изменение	89,6±2,0892,2±5,72,57±5,65	0,7	85,7±2,681,5±2,1-4,15±2,87	0,2
Инсулин (мкЕД/мл)Неделя 0Неделя 8Изменение	10,03±0,910,40±0,80,34±0,82	0,7	9,8±0,99,3±0,71-0,49±0,67	0,5
HOMA-IRНеделя 0Неделя 8Изменение	2,3±0,212,2±0,2-0,05±0,18	0,8	2,11±0,211,9±0,2-0,25±0,18	0,2

Группа иранских ученых продемонстрировала положительные результаты лечения пробиотиками женщин с СПЯ в течение восьми недель. При продолжении терапии у той же когорты женщин до 12 недель было отмечено, что более длительное употребление микробных пищевых добавок также дает хороший метаболический эффект [28].

Что касается потенциального механизма действия пробиотиков, то в работе американских исследователей сообщается, что полезные микробы Bifidobacterium lactis V9 могут усиливать рост микробов Akkermansia, Butyricimonas и Faecalibacterium prausnitzii, продуцирующих короткоцепочечные жирные кислоты. Таким образом, пробиотик Bifidobacterium lactis V9 может положительно влиять на течение синдрома через выработку короткоцепочечных жирных кислот, которые, в свою очередь, влияют на секрецию медиаторов кишечника и мозга, включая грелин и пептид YY [29].

Пребиотики представляют собой неперевариваемые соединения, являющиеся модуляторами роста и/или активности кишечной микробиоты [30]. Они также могут быть использованы в качестве нутритивной поддержки женщин с СПЯ для улучшения метаболических показателей.

Клиническое исследование, проведенное S. Gholizadeh Shamasbi et al. с участием 62 женщин, показало, что потребление резистентного декстрина приводит к снижению содержания в крови маркеров СПЯ: общего холестерина, триглицеридов, холестерина ЛПНП, дегидроэпиандростерона сульфата (ДГЭА-С) и свободного тестостерона, а также положительно влияет на уровень холестерина ЛПВП [31]. Авторы предполагают, что потенциальным механизмом полученного эффекта может быть усиление продукции глюкагоноподобного пептида 1 (ГПП-1) и короткоцепочечных жирных кислот, по-видимому, определенной флорой.

Учитывая вышесказанное, многие исследователи сходятся во мнении, что пробиотики и пребиотики можно рассматривать как эффективные нутритивные средства в дополнение к основным терапевтическим подходам при СПЯ [24].

## РОЛЬ АМИНОКИСЛОТ В ДИЕТОТЕРАПИИ СПЯ

Аминокислоты с разветвленной цепью (Branched-chain amino acids — BCAA) играют важную роль в анаболическом воздействии на массу тела, синтезе мышечного белка и гомеостазе глюкозы. Существует ряд исследований, продемонстрировавших, что как избыточное потребление BCAA, так и их недостаток, связаны с резистентностью к инсулину, ожирением и даже с сахарным диабетом второго типа (СД2). Таким образом, аминокислоты с разветвленной цепью могут быть вовлечены в инициацию развития СПЯ или служить его биомаркерами. Некоторые кишечные микробы могут синтезировать определенное количество BCAA, а нарушение кишечной микробиоты способствует развитию СПЯ через метаболизм аминокислот. Например, W. Chen и Y. Pang выявили, что Prevotella copri способна индуцировать резистентность к инсулину, усугублять непереносимость глюкозы, повышать циркулирующие уровни BCAA и Bacteroides vulgatus, может функционировать как один из основных синтезаторов BCAA в кишечнике человека [6]. Обнаружено также, что Bacteroides vulgatus широко распространен в кишечнике пациенток с СПЯ [32][6].

## РОЛЬ ВИТАМИНА D И КАЛЬЦИЯ В ЛЕЧЕНИИ СПЯ

Витамин D — предиктор инсулинорезистентности, регулятор экспрессии субстрата рецептора инсулина, инсулиноподобного фактора роста, в связи с чем высока актуальность работ, показывающих значимость поддержания адекватного уровня витамина D в крови пациенток с СПЯ. В последние годы проводилось очень большое количество исследований о влиянии витамина D на патогенетические звенья СПЯ. Их результаты не всегда однозначны: при сравнении групп СПЯ с контрольными недостаточность, а тем более дефицит витамина D как правило наблюдается в обеих группах каждого исследования (табл. 4) [33][34].

**Table:** Таблица 4. Уровень витамина D в крови при СПЯ

Численность исследованных женщин	Недостаточность витамина D	Дефицит витамина D	Авторы, год публикации
СПЯ	Контр.	СПЯ	Контр.
N=280 с СПЯ;n=1573 — контроль	45,3%	45,1%	6,3%	8,9%	Lumme J. et al., 2019 г. [33]
N=639 с СПЯ;n=449 без СПЯ	30%	24%	21%	11%	Krul-Poel YHM. et al., 2018 г. [34]

Однако в метаанализе, проведенном в США на основе 30 статей, было показано, что существует связь между дефицитом витамина D и развитием метаболических нарушений у женщин с СПЯ (табл. 5) [35].

**Table:** Таблица 5. Метаболические показатели у пациенток с СПЯ при дефиците и норме витамина D [35]

Лабораторные показатели	Кол-во исслед.	Кол-во наблюд.	SMD(95% ДИ)	Тест на гетерогенность	Предвзятость публикаций
Р	I² (%)	Р
Глюкоза натощак	4	5	0,31(0,10; 0,53)	0,429	0,0	0,254
Инсулин натощак	4	5	0,63(0,42; 0,85)	0,146	41,3	0,077
HOMA-IR	4	5	1,11(0,51; 1,71)	0,002	76,5	0,13
Общий холестерин	3	4	−0,14(−0,67; 0,40)	0,026	67,7	0,765
ХС-ЛПНП	2	3	−0,11(−0,39; 0,16)	0,101	56,3	0,658
Триглицериды	3	4	−0,17(−1,33; 0,99)	<0,001	92,7	0,657
СРБ	3	4	0,12(−0,67; 0,92)	<0,001	85,2	0,757
Общий тестостерон	4	4	0,08(−0,28; 0,60)	0,075	56,5	0,576
ГСПГ	4	4	0,16(−0,28; 0,60)	0,018	70,1	0,656

В нескольких исследованиях сообщалось, что лечение витамином D на фоне его недостаточности может снизить уровень АМГ у пациенток с СПЯ, улучшить ИР и качество эмбрионов после ЭКО [36][37].

Есть данные, что у пациенток, страдающих СПЯ, прием витамина D в дозе 50 000 МЕ в неделю может влиять на выраженность гирсутизма, уровень андрогенов, инсулина натощак, HOMA-IR, холестерина ЛПНП, улучшая эти показатели (табл. 6) [38][39][40].

**Table:** Таблица 6. Методика лечения СПЯ витамином D

Авторы, год публикации	Численность исследованных	Дозировка витамина D, длительность лечения
Shan Guo et al., 2020 г. [38]	13 РКИn=824 женщ. с СПЯ	3200 — 12 000 МЕ/сутили 50 000 МЕ/нед от 8 до 24 нед
Chen-Yun et al., 2020 г. [39]	11 РКИn=483 женщ. с СПЯ	3200 — 12 000 МЕ/сутили 50 000 МЕ/нед от 8 до 24 нед
Al-Bayyari Nahla et al., 2021 г. [40]	N=60 женщ. с СПЯ	50 000 МЕ/нед в течение 12 недель

Витамин D связан также с гомеостазом кальция, который играет важную роль в сигнальном пути инсулина, фолликулогенезе и созревании ооцитов [41]. В исследовании, проведенном еще в 2012 г., приняли участие 100 женщин с СПЯ, которых произвольно разделили на две группы. В первой группе получали терапию метформином 1500 мг/сут, во второй — помимо метформина в той же дозировке + витамин 25(ОН)D 100 000 МЕ/мес + кальций 1000 мг/сут. Перед началом исследования, а также через три и шесть месяцев оценивали ИМТ, регулярность менструального цикла и диаметр фолликулов. Именно во второй группе (с добавлением витамина D и кальция) авторы отметили улучшение показателей фолликулогенеза и коэффициента фертильности (среднее число рождений на одну женщину), хотя результаты не были статистически значимыми [42].

Тем не менее патогенетический механизм влияния дефицита витамина D на течение СПЯ остается предметом дальнейшего изучения. В связи с тем, что различные дозы витамина D могут иметь разные эффекты, для улучшения маркеров СПЯ, как показало исследование Y.H.M. Krul-Poel et al., уровень витамина 25(ОН)D в крови должен быть выше 60 нг/мл [34]. Однако, чтобы персонифицировать эффективную дозу витамина D, необходимы дальнейшие исследования по определению оптимального дозирования каждой пациентки с СПЯ с учетом общего состояния здоровья.

## РОЛЬ ИНОЗИТОЛА В ЛЕЧЕНИИ СПЯ

Инозитол, или витамин В8 — шестиатомный органический спирт (циклогексан), существующий в 9 стереоизомерах (цис-, эпи-, алло-, мио-, нео-, сцилло-, L-хиро-, D-хиро- и мукоинозитол). В живых клетках наиболее распространены миоинозитол (МИ) и D-хироинозитол (D-ХИ). Под действием инсулина МИ конвертируется в D-ХИ при участии фермента тканеспецифических эпимераз, которые, в свою очередь, активируются в ответ на инсулин. Миоинозитол поступает в организм с пищей (проростки, фрукты, бобовые, зерна и орехи), но также может синтезироваться эндогенно из глюкозо-6-фосфата преимущественно в почках [43][44]. В настоящее время МИ и D-ХИ, наиболее изученные изоформы, классифицируются как сенсибилизаторы инсулина. В форме гликанов ключевые ферменты, контролирующие D-хиро-фосфогликан и МИ-фосфогликан, участвуют в метаболизме глюкозы и липидов. В форме фосфоинозитидов они играют важную роль в качестве вторичных посредников в ряде клеточных биологических функций. Обе формы инозитола могут действовать как сенсибилизаторы инсулина и восстанавливать чувствительность к инсулину, так и действовать на яйцеклетку и стероидогенез.

В настоящее время продолжаются исследования, посвященные оценке влияния инозитола на восстановление регулярного менструального цикла, овуляции, снижение степени выраженности гирсутизма и продукции андрогенов у женщин с СПЯ. В последних клинических рекомендациях по СПЯ данную терапию эксперты отнесли к экспериментальной, поскольку наличие на рынке различных форм и дозировок инозитола, а также отсутствие достаточной доказательной базы пока не позволяют сделать окончательный вывод клинической эффективности [3]. Кроме того, не существует единого мнения о том, какая из форм инозитола — МИ, D-ХИ — или их одновременное назначение может оказывать оптимальный терапевтический эффект при СПЯ [45][46][47]. Важно отметить, что препараты практически не вызывают побочных эффектов в физиологических дозах.

Начиная с 2000-х гг., в ряде исследований было показано, что, как МИ, так и D-ХИ, могут улучшить метаболический профиль при СПЯ. Инсулинорезистентность и гиперинсулинемия являются одной из патофизиологических основ этого синдрома, и при дефиците инозитола им может сопутствовать нарушение инозитол-опосредованной передачи сигналов. Учитывая ключевую роль, которую играют инсулинорезистентность и избыток андрогенов у пациентов с СПЯ, инсулиносенсибилизирующие эффекты МИ и D-ХИ были изучены с целью положительного влияния на различные клинические проявления СПЯ, включая возможность восстановления фертильности пациенток [48][49].

В метаанализ, проведенный Unfer V. et al. и посвященный применению МИ при СПЯ, было включено 9 РКИ (247 женщин с СПЯ, получавших МИ, и 249 женщин с СПЯ, не получавших данную терапию (группа контроля) [50]. Назначение МИ в дозе от 500 мг до 1500 мг в день приводило к статистически значимому снижению тощакового уровня инсулина (SMD=−1,021 нг/мл, 95% ДИ: −1,791 −0,251, P=0,009) и индекса инсулинорезистентности НОМА-IR (SMD=−0,585, 95% ДИ: −1,145 до −0,025, P=0,041). Также было отмечено незначительное положительное влияние МИ на средний уровень тестостерона в крови по сравнению с женщинами контрольной группы. При анализе подгрупп было показано значительное повышение уровня ГСПГ среди тех пациенток, которые получали МИ не менее 24 недель (SMD=0,425 nmol/L, 95% ДИ: 0,050–0,801, P=0,026). Авторы делают вывод о том, что МИ оказывает существенный положительный метаболический эффект, улучшая чувствительность к инсулину, что в свою очередь опосредованно может снижать гиперандрогению при СПЯ.

Похожие результаты были получены в исследовании (табл. 7) [51]. В рандомизированное контролируемое исследование было включено 46 пациенток с СПЯ, которых разделили на две группы: 21 пациентка в группе А получала комбинированный препарат МИ+D-ХИ 550 мг+13,8 мг и 25 пациенток в группе В — плацебо. При оценке лабораторных показателей через 6 месяцев постоянного приема препаратов зарегистрировано статистически значимое снижение ЛГ, свободного тестостерона, инсулина натощак, НОМА-IR, а также статистически значимое повышение уровня 17-бета-эстрадиола. Авторы делают вывод, что прием комбинированных форм инозитола эффективен в качестве лечения СПЯ.

**Table:** Таблица 7. Эффективность комбинированной терапии МИ и D-ХИ при лечении СПЯ в течение шести месяцев [51]

Лабораторные показатели (един. измерения)	Группа А (n=21)	Группа В (n=25)
Исходные данные	МИ+D-ХИ	Р	Исходные данные	плацебо	Р
ФСГ (мМЕ/мл)	5,86±1,75	4,96±1,74	NC	5,67±1,11	5,47±0,63	NC
ЛГ (мМЕ/мл)	12,5±8	8,5±4,04	p<0,05	11,27±7,2	11,25±5,35	NC
Эстрадиол (пг/мл)	47,06±18,2	107,42±92,86	p<0,01	50,37±19,45	52±20,2	NC
Инсулин натощак (мЕд/мл)	20,19±8,15	10,74±5,46	p<0,001	18±8	17,8±8,2	NC
Глюкоза натощак (мг/дл)	85±5,96	86±7,12	NC	86,2±9,1	84,73±8,3	NC
Св. тестостерон (нг/дл)	0,76±0,2	0,62±0,15	p<0,05	0,85±0,22	0,83±0,2	NC
ГСПГ (нмоль/л)	24,11±10,35	35,85±24,3	p<0,05	20,44±8,77	21,36±7,57	NC
Андростендион (нг/мл)	4,25±1,48	4,01±1,70	NC	3,48±1,21	3,1±2,23	NC
ДГЭАС (мкг/дл)	327,32±150,89	347,6±170,98	NC	337,95±155,79	315,83±145,59	NC
HOMA-IR	3,38±1,97	1,97±1,48	p<0,05	3,48±2,02	2,8±1,4	NC

Таким образом, в настоящее время МИ рассматривается в качестве потенциального патогенетического лечения СПЯ, сравнимого по эффективности с метформином, но при этом не имеющго побочных эффектов. В то же время изучение роли инозитола в патогенезе заболевания продолжается.

## РОЛЬ ФОЛИЕВОЙ КИСЛОТЫ И ВИТАМИНА В12 В ЛЕЧЕНИИ СПЯ

В 2020 г. в своем исследовании M. Szczuko et al. продемонстрировали, что у женщин с СПЯ ниже уровень фолиевой кислоты и других витаминов группы В, но выше содержание витамина С в крови в сравнении с женщинами без СПЯ и нормальным ИМТ [52].

Принимая во внимание, что ИР является одним из ведущих патогенетических механизмов развития СПЯ, понятен большой интерес исследователей к проблеме повышенного уровня гомоцистеина, избыток которого наблюдается у многих пациенток с СПЯ. Инсулин ингибирует активность цистатионин-бета-синтетазы, что ведет к увеличению концентрации гомоцистеина, являющегося фактором риска развития многих сосудистых осложнений [53]. Витамин В12 необходим для реметилирования гомоцистеина до метионина. В исследовании, проведенном в 2009 г., приняли участие 122 женщины, из них 61 с СПЯ и ожирением; другая половина составила контрольную группу. Было показано, что у женщин с СПЯ наблюдался более высокий уровень гомоцистеина крови и более низкое содержание витамина В 12. Это свидетельствует о непосредственной связи ИР с уровнем гомоцистеина и витамина В 12, что определяет необходимость восполнения витамина В12 при его недостатке или дефиците [54].

При фармакотерапии СПЯ метформином проявляется побочный эффект препарата — нарушение всасывания витаминов группы В, что может приводить к увеличению концентрации гомоцистеина крови. Поэтому применение метформина, который, согласно существующим клиническим рекомендациям, является препаратом 2-й линии терапии СПЯ (таково же мнение исследователей), целесообразно сочетать в течение 12 недель два раза в день с приемом витаминов группы В (В1 — 250 мг; В6 — 250 мг; В12 — 1000 мкг). Такое комплексное лечение оказывает положительное влияние на уровень гомоцистеина [55].

Было доказано, что и отдельный прием фолиевой кислоты в дозировке 5 мг в день является эффективным способом снижения гомоцистеина. В систематическом обзоре литературы в PubMed, Web of Science и EMBASE, а также в предыдущих/систематических обзорах и метаанализах выявлены 29 РКИ (22 250 участников), в которых оценивалось влияние плацебоконтролируемых добавок фолата отдельно или в сочетании с другими витаминами группы B — на глюкозу натощак, инсулин, HOMA-IR, на гликированный гемоглобин и риск развития СД2. По сравнению с плацебо прием фолиевой кислоты снижал уровень инсулина натощак: WMD: -13,47 пмоль/л; 95% ДИ: -21,41; -5,53 пмоль/л. P<0,001. Снизился также уровень HOMA-IR: WMD: -0,57; 95% ДИ: -0,76, -0,37; P<0,0001) [56].

У пациенток с СПЯ и повышенным уровнем гомоцистеина (≥15 мкмоль/л) фолиевая кислота способствует уменьшению массы тела, тогда как у людей с нормальным уровнем гомоцистеина существенного влияния на массу тела фолиевая кислота не оказывает [57].

Итак, добавление в рацион пациенток с СПЯ фолиевой кислоты (5 мг/сут) и витамина В12 (2000 мкг/сут) может оказывать положительный эффект на их метаболические показатели.

## РОЛЬ ВИТАМИНА Е В ЛЕЧЕНИИ СПЯ

Жирорастворимый антиоксидант, витамин Е, играет важную роль в защите клеточной мембраны от окислительного стресса, перекисного окисления, влияет на синтез коллагена. В доказательной медицине используется в лечении дисменореи. Прием витамина Е женщинами с СПЯ улучшает у них уровень глюкозы, липидов и биомаркеров, связанных с андрогенами [58]. Значительное снижение гирсутного числа наблюдалось у пациенток с СПЯ после приема витамина Е (400–900 МЕ/сут) в качестве монотерапии или витамина Е (400–900 МЕ/сут) вместе с омега-3 полиненасыщенными жирными кислотами (омега-3 ПНЖК) (1000 мг/сут) или добавками магния (250 мг) по сравнению с женщинами, принимавшими плацебо [59]. В целом употребление омега-3 ПНЖК (1000 мг/сут) и витамина Е (400 МЕ/сут) в течение 12 недель значительно улучшало липидный профиль и биомаркеры окислительного стресса у женщин с СПЯ (табл. 8) [60].

**Table:** Таблица 8. Эффективность лечения СПЯ витамином Е + омега-3 ПНЖК [60]

Лабораторные показатели (единицы измерения)	Группа плацебо(n=34)	Витамин Е+омега-3 ПНЖК(n=34)
Нед. 0	Нед. 12	Нед. 0	Нед. 12
Триглицериды (мг/дл)	120,6±59,4	128,3±23,6	122,7±61,7	100,6±54,0
Общ. холестерин (мг/дл)	166,4±29,2	178,6±29,9	181,8±28,0	161,5±31,4
ЛПНП (мл/дл)	92,9±26,2	104,8±26,3	11,1±26,5	94,4±29,8
ФСГ (мМЕ/мл)	7,9±2,8	8,1±3,2	7,3±2,5	7,2±2,5
ЛГ (мМЕ/мл)	13,5±13,3	11,4±7,7	11,0±8,0	10,5±8,9

## РОЛЬ ПРЕПАРАТОВ ГПП-1 В ЛЕЧЕНИИ СПЯ

В последние годы агонисты рецепторов глюкагоноподобного пептида-1 (арГПП-1) появились в качестве нового средства терапии СПЯ, поскольку у ГПП-1 обнаружено много уникальных преимуществ для лечения метаболических заболеваний: ингибирование опорожнения желудка и последующих приемов пищи; увеличение секреции инсулина [61]. Многие клинические исследования показали, что арГПП-1 обладают хорошими метаболическими эффектами в отношении снижения массы тела, улучшения ИР и опосредованного снижения уровня андрогенов, что может в дальнейшем значительно расширить показания к их применению при СПЯ [62]. В некоторых исследованиях сообщалось о положительном влиянии арГПП-1 на увеличение частоты наступления беременности посредством ЭКО и частоту естественной беременности у пациенток с ожирением и СПЯ в результате снижения массы тела и нормализации менструального цикла [63][64].

В международном клиническом протоколе по СПЯ (2023 г.) в дополнение к коррекции образа жизни названы препараты для фармакологической помощи пациенткам: лираглутид и семаглутид, а также орлистат [3].

## ЗАКЛЮЧЕНИЕ

СПЯ имеет большое социальное и клиническое значение в связи со значительной распространенностью у женщин фертильного возраста и является одной из наиболее частых эндокринопатий. У каждой 10-й пациентки репродуктивного возраста встречается изучаемый синдром.

Необходимость дальнейшего изучения этиологических и патогенетических путей развития СПЯ определяется высокой социальной значимостью данного заболевания и отсутствием высокоэффективных и безопасных методов его лечения, позволяющих достичь стойкого эффекта в восстановлении регулярного овуляторного менструального цикла, фертильности и устранения симптомов гиперандрогении. В связи с тем, что СПЯ является полигенным эндокринным расстройством, в настоящее время коррекции поддаются только эпигенетические факторы.

При этом подход к лечению рассматриваемого синдрома должен быть персонализированным, учитывающим особенности нутритивного статуса каждой пациентки.

Суммируя проанализированные источники, можно сделать вывод, что диетотерапия и нутритивный статус могут оказывать существенное положительное влияние на маркеры СПЯ, способствуя нормализации метаболических нарушений, восстановлению овуляторной дисфункции, снижению продукции андрогенов.

Распространенность синдрома в мире подчеркивает важность многофакторного и комплексного подхода к его лечению, а также необходимость дальнейших поисков новых методов терапии синдрома поликистозных яичников.

## ДОПОЛНИТЕЛЬНАЯ ИНФОРМАЦИЯ

Источник финансирования. Работа выполнена в рамках государственного задания №123021300169-4 «Эпигенетические предикторы и метаболомная составляющая аменореи различного генеза у женщин репродуктивного возраста», 2023–2025 гг.

Конфликт интересов. Авторы декларируют отсутствие явных и потенциальных конфликтов интересов, связанных с содержанием настоящей статьи.

Участие авторов. Все авторы одобрили финальную версию статьи перед публикацией, выразили согласие нести ответственность за все аспекты работы, подразумевающую надлежащее изучение и решение вопросов, связанных с точностью или добросовестностью любой части работы.
